# Vaccination Coverage of People Living with HIV: Before and after Interventional Action

**DOI:** 10.3390/vaccines12080897

**Published:** 2024-08-08

**Authors:** Larissa Gerin, Elucir Gir, Lis Aparecida de Souza Neves, Luzia Márcia Romanholi Passos, Renato de Ávila Kfouri, Bruno Spire, Renata Karina Reis

**Affiliations:** 1 Epidemiological Surveillance Division, Ribeirão Preto Municipal Health Department, Ribeirão Preto 14015100, São Paulo, Brazil; lisapneves@yahoo.com.br (L.A.d.S.N.); lmrpassos@rp.ribeiraopreto.sp.gov.br (L.M.R.P.); 2 Ribeirão Preto School of Nursing, University of São Paulo, Ribeirão Preto 14040902, São Paulo, Brazil; egir@eerp.usp.br (E.G.); rkreis@eerp.usp.br (R.K.R.); 3 Brazilian Society of Immunization, São Paulo 01309902, São Paulo, Brazil; renatokfouri@uol.com.br; 4 Inserm, IRD, SESSTIM, Sciences Economiques & Sociales de la Santé & Traitement de l’Information Médicale, ISSPAM, Aix Marseille University, 13385 Marseille, France; bruno.spire@inserm.fr

**Keywords:** vaccination coverage, vaccination, HIV, health professionals

## Abstract

This is a quasi-experimental study that assessed PLHIV vaccination coverage before and after health professionals participated in a training course on PLHIV immunization. The vaccination coverage of 645 PLHIV was assessed in the pre-intervention phase. The vaccine with the best coverage was diphtheria and tetanus (82.64%) and the one with the lowest rate of adequately vaccinated was measles, mumps, and rubella (38.27%). Individuals aged between 30 and 39 years had a 74.00% (1–0.26) lower chance of having the full vaccination schedule when compared to those aged between 10 and 19 years, and among those over 40 years, the chance was 87.00% (1–0.13) lower. Those who were vaccinated in Specialized Care Services (SCS) were 5.77 times more likely to be adequately vaccinated when compared to those who were vaccinated in other health services. Regarding the entire vaccination schedule evaluated, the number of adequately vaccinated increased from 47 (7.29%) to 76 (11.78%). Interventions targeting health professionals were effective in increasing vaccination coverage among PLHIV; however, the achieved coverage remained below the desired level. It is necessary to act on health professionals’ knowledge and other aspects to effectively increase vaccination coverage.

## 1. Introduction

With advances in immunization and improved hygiene and health conditions, a decrease in the number of infectious diseases has been observed; however, the reduction in vaccination coverage and population displacement in the globalized world pose a risk of the resurgence of diseases that have already been eliminated [1].

People living with HIV (PLHIV) have an increased risk of acquiring infectious diseases and, if they do, have a greater chance of developing into more severe forms [2,3,4,5,6,7].

On the other hand, with increased survival and reduced mortality due to adequate adherence to Antiretroviral Therapy (ART), they end up exposing themselves more to risk scenarios as they maintain work, travel, and leisure activities that put them in contact with potential pathogenic infectious agents. In addition, these individuals end up being more exposed as they are in frequent contact with health services [3,8,9,10]. 

Thus, preventing the occurrence of infectious diseases in PLHIV is a strategy to improve the quality of life and life expectancy. In this regard, immunization stands out, and a special vaccination schedule is recommended for these individuals [1,3,10,11,12].

In Brazil, the Unified Health System (UHS) offers 12 immunobiological agents to PLHIV free of charge through the National Immunization Program (NPI). The vaccination schedule for this group is broader than that offered to the general population. In addition, it is recommended that the vaccination schedule of household contacts of PLHIV and health professionals who provide care for this population be updated, which constitutes extra protection, especially for those with some contraindications to vaccination for having lower CD4 T-lymphocyte (TL) counts. It is recommended that the vaccination schedule be updated as early as possible before the disease progresses and the immune system becomes deficient [3,4,8,10,13,14,15,16].

Despite the importance of immunization, few studies have presented vaccination coverage for PLHIV. The majority of studies focus on the vaccination rate for a single vaccine type [2,6,17,18] or inactivated vaccines indicated for this population [1,5,7,9,12,13,19,20], rather than providing a comprehensive overview of vaccination coverage, including attenuated vaccines [3,15]. 

In a survey conducted in Germany on PLHIV aged at least 50 years, only 20% of the participants reported a completed schedule for the inactivated vaccines evaluated [9]. In an investigation conducted in Brazil regarding attenuated vaccines, it identified coverage of 14.14% [3]. In a retrospective cohort study conducted in the USA in which the schedule of eight vaccines indicated for PLHIV was evaluated, it was found that 41% of the participants were adequately vaccinated [19].

The data show that vaccination coverage among PLHIV is low and that there is no effective assessment of vaccination status in the services where these individuals are monitored. The main determinants of non-vaccination are failure to receive a recommendation from a health professional, lack of knowledge about the indicated vaccines and their schedules, difficulties in accessing immunizers, or simply forgetting appointment dates [1,2,3,4,5,6,7,9,11,12,13,15,21].

The set of aspects exposed reinforces the essential role of health professionals in the indication of immunizers, deconstruction of false contraindications, expansion of vaccine acceptance, and search for unvaccinated individuals to improve adequately vaccinated rates. In addition, the importance of medical professionals in vaccine indication and prescription is highlighted, which would significantly increase the search of individuals for immunobiological agents [3,4,6,7,9,12,13,21]. 

Given this scenario, this study aimed to evaluate the effectiveness of an interventional action in the vaccination coverage of PLHIV, considering the vaccines recommended by the Brazilian NIP.

## 2. Materials and Methods

### 2.1. Study Design and Site

The study was conducted in a Brazilian municipality with a population of about 711,000 inhabitants and a Human Development Index (HDI) of 0.800, which is considered high relative to the whole country. The municipality has a wide network, with 47 Primary Health Care Units (PHCU) (37 of them with vaccine rooms) and five Specialized Care Services (SCS), for outpatient clinical follow-up of PLHIV [22]. 

This is a quasi-experimental study conducted in three stages:

- Pre-intervention phase—was developed using data obtained from the Information System for Notifiable Diseases (ISND), which is the national country’s HIV case notification system. These cases were then identified in the municipal outpatient follow-up system (Hygiaweb) in order to assess the records of vaccines administered and calculate vaccination coverage.
- Intervention phase—a training course on immunization of PLHIV was offered to health professionals involved in vaccination actions in the public services of the municipality of the study and to SCS health professionals.
- Post-intervention phase—one year after the start date of the training course in the intervention phase, data on vaccines administered to PLHIV were collected again, updating the information collected in the pre-intervention phase regarding vaccination coverage rates.

### 2.2. Population and Sample

In the pre-and post-intervention phases, the population consisted of all cases notified in the ISND between 2015 and 2020 of people aged at least 13 years who met the following inclusion criteria: being followed up at the SCS of the municipality with the last medical consultation less than 12 months before the start of data collection, residing in the municipality of the study, and having a diagnosis of HIV status up to 180 days from the date of notification. Individuals who died or were transferred to other services or municipalities were excluded. 

In the intervention phase, the population consisted of 77 health professionals (nursing assistants, technicians, and nurses) who were working in the 38 public vaccine rooms of the municipality and the health professionals (nursing assistants, technicians, nurses, and doctors) of the five SCS. The professionals completed an online training course, with four modules and a total workload of 3 h, on issues related to PLHIV immunization.

The course was developed by the researcher, and before it was made available to the research subjects, it underwent a pilot evaluation by six professionals who tested its functionality.

### 2.3. Data Collection

In the pre-and post-intervention phases, data collection was performed on the REDCap platform using a form developed for this study. The form contained sociodemographic variables (sex, age, pregnancy, skin color, education, occupation, and health district of residence) and clinical-epidemiological variables (date and unit of notification, date of diagnosis of HIV infection, follow-up status, date of first and last medical consultation at the service, number of medical consultations, service that performs the follow-up, exposure category, social vulnerabilities, presence of other diseases, CD4 TL count, viral load quantification, use of ART, registered vaccines, and anti-HAV IgG and anti-HBs serology results), and was validated by four specialists and modified according to suggestions received.

Assessing the vaccination schedule is complex, as it requires verification of each vaccine received throughout life, and the schedules can be different if started before or after the diagnosis of HIV infection. In addition, it is necessary to assess susceptibility status in some cases in order to verify whether immunobiological agents are indicated. To ensure an accurate assessment of each individual’s vaccination status, data were collected by a professional with over 20 years of experience in the field.

### 2.4. Data Analysis

In the pre-and post-intervention phases, data were extracted from the REDCap (https://redcap.eerp.usp.br/, accessed on 26 July 2022) platform using a Microsoft Office Excel v. 2406 spreadsheet. After evaluation and correction of possible typing errors, the data were transported to the IBM^®^ Statistical Package for the Social Sciences (SPSS^®^) Statistics version 25 and R i386 v.3.4.0 programs where the database was structured [23]. Data analysis was performed using descriptive statistics (mean and standard deviation for continuous variables) and proportions for categorical variables.

The comparison between the pre-and post-intervention phases was evaluated using McNemar’s test, with *p*-values < 0.05 considered statistically significant. A logistic regression model, using the Generalized Estimating Equations (GEE) method, was used to estimate the variables associated with an adequate vaccination schedule in the post-intervention phase, and the crude and adjusted odds ratio was calculated with 95% intervals for each variable and a significance level of 5%.

The outcome variable was the complete vaccination schedule according to the vaccination schedule recommended by the NIP (Table 1).

### 2.5. Ethical Aspects

The research project was submitted to the Research Project Evaluation Committee (RPEC) of the Municipal Health Department of the studied municipality and later to the Research Ethics Committee (REC) of the Ribeirão Preto Nursing School (RPNS) of the University of São Paulo (USP), obtaining a favorable opinion (REC consolidated opinion No. 4.782.341).

## 3. Results

In the pre-intervention phase, the records of 1688 PLHIV were evaluated. After analyzing the inclusion and exclusion criteria, the sample consisted of 645 individuals in the pre- and post-intervention phases, most of whom were male (83.41%), white (59.84%), and with education above high school (55.97%). The mean age was 32.06 years (SD ± 11.14), with the minimum age being 14 years and the maximum 72 years. The age group with the highest frequency was 20–29 years (45.89%) (Table 2).

**Figure 1:**
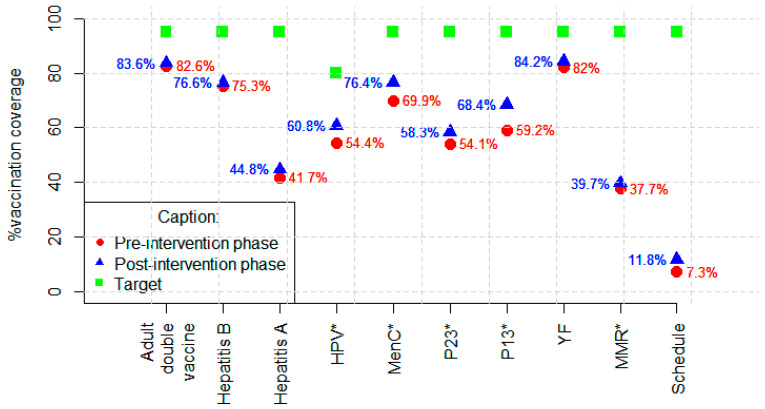
Vaccination coverage by vaccine and schedule were evaluated before and after the intervention. * *p* < 0.005; Adult double vaccine (diphtheria and tetanus); HPV—human papilloma virus; MenC—meningococcal C; P23—23-valent pneumococcal; P13—13-valent pneumococcal; YF—yellow fever; MMR—measles/mumps/rubella.

For diphtheria and tetanus, hepatitis B, and hepatitis A vaccines, although the number of adequately vaccinated increased in the post-intervention phase, the increase was not statistically significant. In the pre-intervention phase, 533 (82.64%) PLHIV were adequately vaccinated against diphtheria and tetanus, 445 (75.30% of those with vaccination indications) against hepatitis B, and 143 (42.43% of those with vaccination indications) against hepatitis A. They were adequately vaccinated in the post-intervention phase: 539 (83.57%) against diphtheria and tetanus, 453 (76.65% of those with vaccine indications) against hepatitis B, and 151 (44.81% of those with vaccine indications) against hepatitis A. Individuals who were not susceptible to the disease were not considered for assessment of adequately vaccinated against hepatitis A and B (Table 3, Figure 1).

In the post-intervention evaluation where the information regarding the tests was updated, 471 (73.02%) individuals had reactive anti-HBs serology; however, for 32 (4.96%), there was no result of this test in the electronic medical record, despite being a recommended test for PLHIV. Regarding hepatitis A, 308 (47.75%) had reactive anti-HAV IgG and were, therefore, not susceptible to the disease without an indication for vaccination. For 112 (17.36%), there was no record of this test in the electronic medical record.

As for the 13-valent pneumococcal vaccine, in the post-intervention phase, 59 (22.43%) of the inadequately vaccinated became adequately vaccinated, and the coverage increased from 59.22% to 68.37%. For the 23-valent pneumococcal vaccine, the number of adequately vaccinated increased from 349 (54.11%) to 376 (58.29%). The number of adequately vaccinated with meningococcal C increased from 451 (69.92%) to 493 (76.43%). For the HPV vaccine, among those with an indication for vaccination according to the recommendation at the time of data collection, coverage increased from 57.02% to 62.55% (Table 3, Figure 1).

For attenuated vaccines, which are contraindicated in the presence of severe immunodepression, coverage of the measles/mumps/rubella vaccine (triple viral) increased from 38.27% to 39.69%, and for the yellow fever vaccine, coverage increased from 82.66% to 84.22% (Table 3, Figure 1). Individuals who had a last CD4 LT count of <200 cells/mm^3^ were excluded from the analysis in the post-intervention phase because they had contraindications for these vaccines.

Regarding the entire vaccination schedule evaluated according to the study proposal, the number of adequately vaccinated increased from 47 (7.29%) to 76 (11.78%) (Table 3, Figure 1).

In the multivariate analysis of factors associated with vaccination rates, the model results indicate that as age increases, the chance of having a complete vaccination schedule decreases. Individuals aged between 30 and 39 years had a 74.00% lower chance (adjusted OR 0.26 95% CI 0.09–0.74) of having a complete vaccination schedule when compared to those aged between 10 and 19 years, and among those over 40 years, this rate was 87.00% lower (adjusted OR 0.13 95% CI 0.03–0.50) (Table 4).

Individuals who were followed up at SCS 3 had a 67.00% (adjusted OR 0.32, 95% CI 0.11–0.91) lower chance of being adequately vaccinated when compared to individuals who were followed up at SCS 2. Those with good adherence to ART were 5.17 times (95% CI 1.16–22.97) more likely to have a complete vaccination schedule when compared to individuals who did not have good adherence. Those who were vaccinated in SCS were 5.76 (95% CI 1.65–20.19) times more likely to be adequately vaccinated when compared to those who were vaccinated in other health services (Table 4).

Individuals with some social vulnerability were 2.3 times (95% CI 1.06–4.99) more likely to be adequately vaccinated when compared to those with no identified vulnerability. In the assessment performed after the intervention phase, individuals were 1.72 (95% CI 1.39–2.13) times more likely to have the complete vaccination schedule when compared to the pre-intervention phase. There was no association between the complete vaccination schedule and the variables sex, skin color, CD4 TL count, and exposure category (Table 4).

## 4. Discussion

In this study, a very small proportion of PLHIV had an appropriate vaccination schedule according to the proposed assessment, both pre- and post-intervention, which reinforces that there is no effective assessment of the vaccination situation in the services that accompany PLHIV [12]. After offering a training course for health professionals from vaccination rooms and health services that accompany PLHIV, there was an improvement in vaccination coverage, but it still remained low.

Studies that analyze predominantly inactivated vaccines in the evaluation of the vaccination schedule of PLHIV present a higher rate of adequately vaccinated individuals [9,12,19]. In a study that included yellow fever and measles-mumps-rubella vaccines among the vaccines evaluated, only 14.1% of PLHIV were adequately vaccinated, a coverage close to that found in the evaluation performed after this study’s intervention phase [3].

Despite the risk of exposure to vaccine-preventable diseases, there is fear among both health professionals and PLHIV themselves regarding the safety of vaccine administration and the effectiveness of immunobiological agents in this population [2,24]. 

The vaccine with the best coverage was diphtheria and tetanus, as in other studies [5,9,12]. This vaccine has been part of the Brazilian immunization schedule for many years, is available in all vaccine rooms, and presents the same vaccination schedule for the entire population, with few contraindications, which perhaps provides teams with greater safety in administrating this immunobiological agent [12].

The hepatitis B vaccine is administered to people who have never been exposed to the virus or have received the vaccine. In our study, around 25% of the individuals eligible to receive the hepatitis B vaccine were not adequately vaccinated. Our findings are similar to the vaccination rates identified in some studies [3,9,15,20], while the number of people adequately immunized with the hepatitis B vaccine was lower in other reports [5,7,12,13,25]. In Brazil, the hepatitis B vaccine has been part of the national calendar since 1998; therefore, because this study population is mostly young, the rate of individuals without any dose of this vaccine was 2.48% in the post-intervention phase.

The increase in the rate of adequately vaccinated for diphtheria/tetanus and hepatitis B vaccines in the post-intervention phase of this study was small and not statistically significant, which can be explained by the fact that most individuals had already received the full course of these vaccines when they were diagnosed with HIV.

Among the inactive vaccines, the hepatitis A vaccine demonstrated the poorest coverage, even in the post-intervention phase, with an increase of only 3% in the number of individuals adequately vaccinated. Some studies have identified better vaccination rates against hepatitis A than our findings [7,9,20]. However, our coverage was higher than that reported in previous studies on PLHIV, in which vaccine hesitancy was identified as one of the factors for non-adherence to vaccination, in addition to low schooling and immunodepression [5,12,25]. 

Vaccine hesitancy is a complex and dynamic behavioral phenomenon that has been advancing worldwide. It is influenced by confidence in the safety and efficacy of vaccines, the low-risk perception of the population, and because the vaccine is often not available or affordable [26,27,28].

The vaccination coverage of the 13-valent pneumococcal vaccine was around 68% in the post-intervention phase, while that of the 23-valent was around 58%. Some studies identified higher coverage [9,13,20,29], while others identified lower coverage [3,12,15,25]. A study conducted in Germany revealed that 58.5% of the population had received both the 13-valent and 23-valent pneumococcal vaccines, while 11% had received only the 23-valent vaccine [1].

For the two pneumococcal vaccines indicated for PLHIV, there was a significant increase in the number of adequately vaccinated in the post-intervention phase, with 13-valent showing the greatest increase among the vaccines evaluated. 

In an intervention carried out through the implementation of a hospital consultation to assess vaccination status in Spain, the coverage of the 13-valent pneumococcal vaccine increased from 2.9% to 88.0%, and the coverage of the 23-valent pneumococcal vaccine increased from 16.3% to 83.7% [18]. These data reinforce that actions aimed at increasing knowledge and, consequently, confidence in immunizers, targeting both health professionals and patients, have a positive impact on vaccination coverage.

Pneumococcal vaccines and hepatitis A vaccines are not available in vaccination rooms in the studied municipality and need to be requested by filling out a specific form addressed to the Special Immunobiological Agents Reference Center (SIARC). For some months during the study period, pneumococcal vaccines were available in vaccination rooms due to a temporary expansion of indications, which may have contributed to the increase in the number of adequately vaccinated. 

The meningococcal C vaccine was the vaccine with a special indication that showed the best coverage in this study, with an increase of 6.51% of adequately vaccinated in the post-intervention phase. One of the factors that contributed to this rate is the fact that the immunobiological agent is available in vaccination rooms. Lower coverage of the meningococcal vaccine has been identified in other studies [9,12]. Vaccine hesitancy and the unavailability of the immunobiological agent in vaccination rooms contribute to low coverage [2,12,16,21,25,29].

Regarding the HPV vaccine, our study exhibited the most comprehensive coverage among the evaluated studies. Given the recent introduction of the vaccine in the medical landscape, vaccination coverage varies considerably across studies, contingent on the timeframe in which they were conducted. The importance of publicizing the necessity of this vaccine for PLHIV has been reinforced by researchers, given that the transmission route of HPV is the same as that of HIV. This publicizing is of particular significance to the population of men who have sex with men (MSM), given their elevated susceptibility to both acquiring the virus and developing persistent infections and malignant lesions [1,14,30,31,32]. 

On the other hand, the indication of different schemes for different age groups and constant changes in the vaccination scheme can be a factor that confuses teams and contributes to missed vaccination opportunities. Despite this, in the post-intervention phase, the number of adequately vaccinated showed an increase of about 6.00%, which reinforces that more trained teams regarding the indicated schemes favor the improvement in vaccination rates [6].

In order to assess the indication of attenuated vaccines for PLHIV, it is necessary to evaluate the value of the CD4 TL count since severely immunosuppressed individuals are temporarily contraindicated to receive these vaccines [4,14]. As this is a complex assessment and is not always possible, most studies assessing vaccination coverage do not include the evaluation of attenuated vaccines. 

Studies have shown that a large number of PLHIV lack immunity against measles, mumps, and rubella and have reinforced the risk of immunity loss over the years [33,34,35,36,37]. 

In this study, the measles-mumps-rubella vaccine was the one with the worst coverage among the vaccines evaluated, with a small increase in adequately vaccinated individuals in the post-intervention phase despite the low number of individuals with contraindications. In another study conducted in Brazil, 83.8% of PLHIV had not received any dose of measles, mumps, or rubella vaccine [3]. It is important to ensure a complete vaccination schedule to correct possible vaccine failures and maintain seroconversion. 

As the yellow fever vaccine has been part of the vaccination schedule of the studied municipality for many years, due to the fact that it is considered a risk area for the disease, this vaccine coverage was around 84% in the post-intervention phase. Most likely, most of these individuals were already adequately vaccinated before the diagnosis of HIV infection. In addition, the yellow fever vaccination schedule for PLHIV without severe immunodepression does not differ from the schedule indicated for the general population, which is not the case for measles, mumps, and rubella vaccines.

Some authors suggest that vaccination with PLHIV be delayed until the immune system is rebuilt, which would ensure a better response and lower the risk of events supposedly attributable to vaccination or immunization (ESAVI). Although the administration of some vaccines may generate a transient increase in viral load, these events do not present clinical significance, and their risk cannot prevent vaccination [4,9,11,12,15,19,28,38,39].

In this study, individuals with good adherence to ART had a greater chance of being adequately vaccinated, which may indicate better self-care and, in addition, greater confidence on the part of the team in indicating and administering vaccines in this population.

Even if the response to vaccination is reduced for individuals with uncontrolled viral replication or lower CD4 TL counts, it is recommended that the vaccination schedule be updated as soon as possible, taking into account contraindications to attenuated vaccines, without the need for intervals between vaccine administration and test collection [4,9,11,14,38]. A positive approach by the healthcare team in this regard would reduce the number of non-vaccinated [35].

Most of the individuals evaluated in this study were vaccinated in the SCS, which reinforces that having a vaccine room in the services where the individual performs the follow-up facilitates access and increases adherence, and the teams that work in the vaccine rooms of the SCS may be more prepared and safer to administer vaccines to PLHIV [12,15,21]. 

The assessment of vaccination status and guidance on immunization should be part of the medical consultation, with the prescription of vaccines included in the routine of care, and, in addition, be the practice of various health professionals who assist PLHIV in different services [2,3,6,7,12,13,21,24,40,41]. 

It is necessary to verify that the individuals referred for vaccination have actually carried out the procedure, and if they do not appear in the vaccination room or delay the completion of the vaccination schedule, it is extremely important to actively search for these individuals. 

For PLHIV, the evolution of vaccine-preventable diseases may be more severe, and vaccine immunogenicity may be lower, with shorter protection time when compared to people not living with HIV. For this reason, active surveillance of vaccination status is important. In addition, managers need to know the reasons for non-vaccination and develop strategies to combat vaccine hesitancy and improve vaccination rates [6,16,42,43,44,45,46,47,48].

Some limitations should be considered when analyzing these findings, as this study evaluated secondary data through information systems. Without access to individuals, it was not possible to evaluate their vaccination records. Despite the guidance that all vaccination history should be recorded in the system, it is possible that for some individuals, the doses received in other municipalities have not been recorded. The importance of future studies that perform this evaluation with subjects is reinforced. Despite this limitation, this study makes an important contribution to understanding the low vaccination coverage in PLHIV.

## 5. Conclusions

In recent years, Brazil has experienced a drop in vaccination coverage, the causes of which are multifactorial. Intervening in the knowledge of health professionals regarding issues related to the vaccination of PLHIV has enabled an increase in vaccination coverage among this population, but coverage has still remained low.

This study’s findings demonstrate the need to develop measures to improve vaccination rates in populations at a higher risk for vaccine-preventable diseases. Health professionals play a key role in identifying unvaccinated individuals. By recommending vaccination and combating false contraindications, they increase the likelihood that individuals will seek immunization services. 

In order to achieve the desired vaccination coverage rates, in addition to the knowledge of health professionals, it is necessary to act on other factors, such as immunobiological agent availability in the services where PLHIV is monitored. It is also necessary for health managers to invest in addressing vaccine hesitancy.

Improvements in the completeness of vaccination schedules would be enhanced by the introduction of an immunization program in the place where clinical follow-up is carried out. This program could adopt an individualized approach, with doctors and nurses assessing vaccination status, providing advice on the vaccines indicated, prescribing the necessary vaccines, and administering them in the same facility.

## Figures and Tables

**Table 1:** Vaccines included in the evaluation of the complete vaccination schedule according to the recommendations of the National Immunization Program (NIP) at the time of the study.

Vaccine	Recommended Schedule ^†^	Schedule Considered
Adult double (diphtheria/tetanus)	Three doses and boosters every 10 years	Complete or in progress without delays
Hepatitis B	Three doses (before diagnosis) Four doses with double dose (after diagnosis) (for susceptibles)	Complete or in progress without delay
Hepatitis A	Two doses (for susceptible)	Complete or in progress without delay
Viral triple (measles, mumps, rubella)	Two doses	Complete or in progress without delay
Yellow fever	Single dose from 5 years of age or two doses	Complete or in progress without delay
Pneumococcal 23-valent	Two doses	1 dose
Pneumococcal 13-valent	Single dose	1 dose
Meningococcal C	Two doses and booster every 5 years	Complete or in progress without delay
HPV	Three doses (men aged 9–26 and women aged 9–45) ^‡^	Complete or in progress without delay

^†^ Source: [15]. ^‡^ The pre-intervention phase started on 1 August 2021, and the vaccination schedule indicated by the NIP until this date was considered in the evaluation, without incorporating subsequent updates.

**Table 2:** Distribution of people living with HIV/AIDS according to sociodemographic variables.

Sociodemographic Variables	Participants	
	n	%
Sex		
Male	538	83.41
Female	107	16.59
Skin color		
White	386	59.84
Black	65	10.08
Yellow	3	0.47
Brown	169	26.20
Ignored	22	3.41
Level of education		
Illiterate or incomplete primary education	13	2.02
Complete primary school	69	10.70
Complete elementary school	97	15.04
Complete high school	278	43.10
Complete higher education	83	12.87
Ignored	105	16.28
Age group (years)		
13 to 19	39	6.05
20 to 29	296	45.89
30 to 39	173	26.82
40 to 49	74	11.47
50 to 59	45	6.98
60 or more	18	2.79

**Table 3:** Registered vaccination schedules for people living with HIV/AIDS in the pre- and post-intervention phases.

Study Variables	Number of Participants with an Indication for the Vaccine		Pre-Intervention		Post-Intervention		*p*-Value
	n	%	Complete	Incomplete	Complete	Incomplete	
Adult double vaccine	645	100	533	112	539	106	0.480
Hepatitis B	590	91.47	445	145	453	137	0.341
Hepatitis A	335	51.94	142	193	150	185	0.118
13-valent pneumococcal	645	100	382	263	441	204	**<0.001**
23-valent pneumococcal	645	100	349	296	376	269	**0.040**
Meningococcal C	645	100	451	194	493	452	**<0.001**
HPV	234	36.28	134	100	147	87	**0.002**
MMR	635	98.45	243	392	252	383	**0.016**
Yellow Fever	640	99.22	529	111	539	101	**0.004**
Vaccination schedule	645	100	48	597	75	570	**<0.001**

Adult double vaccine (diphtheria and tetanus); HPV—human papillomavirus; MMR—measles/mumps/rubella. Bolded values are significant at 0.05 level.

**Table 4:** Multivariate analysis of factors associated with vaccination coverage of people living with HIV/AIDS.

Study Variables	Number of Participants		Crude OR (95% CI)	*p*-Value	Adjusted OR (95% CI)	*p*-Value
	n	%				
Sex						
Male	538	83.41	REF ^†^		REF ^†^	
Female	107	16.59	0.16 (0.05–0.55)	**0.004**	0.44 (0.12–1.55)	0.201
Skin color ^‡^						
White	386	59.84	REF ^†^		REF ^†^	
Black	65	10.08	0.36 (0.1–1.23)	0.103	0.47 (0.14–1.65)	0.241
Mixed race/Asian	172	26.67	0.99 (0.56–1.73)	0.963	1.03 (0.56–1.89)	0.927
Age group (years)						
13 to 19	39	6.05	REF ^†^		REF ^†^	
20 to 29	296	45.89	0.67 (0.31–1.45)	0.307	0.56 (0.23–1.34)	0.193
30 to 39	173	26.82	0.27 (0.11–0.69)	**0.006**	0.26 (0.09–0.74)	**0.011**
40 and over	137	21.24	0.1 (0.003–0.37)	**0.001**	0.13 (0.03–0.51)	**0.003**
Exposure category ^‡^						
Non-heterosexual	397	61.55	REF ^†^		REF ^†^	
Heterossexual	223	34.57	0.22 (0.1–0.47)	**0.001**	0.53 (0.23–1.22)	0.134
Unit where follow-up is carried out						
Specialized Care Service 2 ^§^	190	29.46	REF ^†^		REF ^†^	
Specialized Care Service 1 ^‖^	124	19.22	0.47 (0.2–1.08)	0.075	0.44(0.19–1.01)	0.052
Specialized Care Service 3 ^‖^	98	15.20	0.29 (0.11–0.82)	**0.019**	0.33 (0.12–0.91)	**0.033**
Specialized Care Service 4 ^‖^	109	16.90	1.12 (0.57–2.2)	0.746	1.24 (0.62–2.51)	0.542
Specialized Care Service 5 ^¶^	124	19.22	0.86 (0.43–1.73)	0.676	1.23 (0.58–2.59)	0.591
CD4 T-lymphocyte count (cells/mm3) ^‡^						
All > 350	115	17.83	REF ^†^		REF ^†^	
Some < 200	96	14.88	0.34 (0.14–0.78)	**0.011**	0.64 (0.27–1.49)	0.296
Some between 200 and 350 (none < 200)	433	67.13	0.71 (0.33–1.52)	0.373	0.86 (0.41–1.84)	0.704
Use of Antiretroviral Therapy ^‡^						
No	104	16.2	REF ^†^		REF ^†^	
Yes	539	83.2	7.64 (1.72–34.02)	**0.008**	5.17 (1.17–22.97)	**0.031**
Vaccine room where patients received the last dose of vaccine						
Other units	105	16.28	REF ^†^		REF ^†^	
Specialized Care Service	540	83.72	6.59 (1.92–22.68)	**0.003**	5.77 (1.65–20.19)	**0.006**
Social Vulnerability						
No	489	75.81	REF ^†^		REF ^†^	
Yes	156	24.19	2.3 (1.09–4.85)	**0.029**	2.3 (1.06–4.99)	**0.034**
Complete vaccination schedule						
Pre-intervention phase	47	7.29	REF ^†^		REF ^†^	
Post-intervention phase	76	11.78	1.62 (1.33–1.96)	**0.001**	1.72 (1.39–2.13)	**0.001**

^†^ Reference group; ^‡^ Data such as “unknown”, “examination not available”, “no information” were not taken into account for statistical processing; ^§^ Specialized Care Service, which had a vaccine room; ^‖^ Specialized Care Services, which operated in the same facility of basic units with vaccine rooms; ^¶^ Specialized Care Service, which did not have a vaccine room in the facility. Bolded values are significant at 0.05 level.

## Data Availability

Data from this study can be made available upon request.
